# Getting de Pointes Across: Isoproterenol for Refractory Methadone-Associated Torsades de Pointes in Alcohol Withdrawal

**DOI:** 10.7759/cureus.100838

**Published:** 2026-01-05

**Authors:** Jennifer Wrona, Carmelita Coca, Tyson Dietrich, Kesoma Holcomb

**Affiliations:** 1 Pharmacy Services, Banner Health, Phoenix, USA; 2 Pharmacy, Christus St. Vincent Regional Medical Center, Santa Fe, USA; 3 Pharmacy/Infectious Diseases, Kingman Regional Medical Center, Kingman, USA; 4 Pharmacy/Behavioral Health and Pain Management, Kingman Regional Medical Center, Kingman, USA

**Keywords:** drug-induced qtc prolongation, drug-induced torsades de pointes, methadone-associated injury, methadone toxicity, qtc prolongation, torsade de pointes

## Abstract

Methadone is known to prolong the QTc interval and may precipitate torsades de pointes (TdP), particularly at higher doses and in the presence of additional risk factors. Standard management involves discontinuation of the offending agent, correction of electrolyte imbalances, and administration of intravenous magnesium; however, some patients may continue to experience recurrent TdP. We present a case of a young female on high-dose methadone, who developed TdP in the context of acute alcohol withdrawal and clonidine-associated bradycardia. Her arrhythmia persisted despite treatment with magnesium and lidocaine but resolved following initiation of an isoproterenol infusion. This case emphasizes the importance of systematic risk assessment in patients receiving methadone and supports the use of isoproterenol as a viable therapeutic option when TdP is refractory to first-line interventions.

## Introduction

Methadone is a long-acting µ-opioid receptor agonist used for both opioid use disorder and chronic pain management. Its prolonged duration of action allows once-daily dosing in maintenance programs and attenuates withdrawal symptoms during opioid tapering. However, methadone exhibits highly variable pharmacokinetics and has been associated with dose-related QTc prolongation and torsades de pointes (TdP). Several case series and observational reports have described significant QTc prolongation, TdP, and sudden cardiac death in patients receiving therapeutic and high-dose methadone, particularly in the presence of additional predisposing factors. This has led to a growing emphasis on careful risk assessment and electrocardiogram (EKG) surveillance in methadone-treated patients, while underscoring the practical challenges of balancing its substantial benefits against the risk of rare but potentially fatal ventricular arrhythmias [1-3].

Patients are at increased risk for QTc prolongation due to several factors, including advanced age, female sex, electrolyte disturbances, bradycardia, underlying heart disease, and the use of concurrent QTc-prolonging medications [2]. QTc is generally considered prolonged when it exceeds 450 milliseconds (ms) in men and 460 ms in women [2]. The risk of developing TdP is highest when the QTc exceeds 500 ms or increases abruptly by more than 40 ms [2].

Torsades de pointes, French for “twisting of the points,” is a polymorphic ventricular tachycardia characterized by QRS complexes that twist around the isoelectric line on the EKG [3-5]. Although TdP may terminate spontaneously, it can also degenerate into ventricular fibrillation, potentially resulting in sudden cardiac death if not promptly treated [3-5]. Acquired forms of TdP are typically triggered by inhibition of the delayed rectifier potassium current (IKr), which prolongs repolarization and increases susceptibility to early afterdepolarizations that may initiate the arrhythmia [3-5].

Initial management focuses on reducing modifiable risk factors, including discontinuation of nonessential QTc-prolonging medications and correction of electrolyte imbalances [3-5]. In hemodynamically unstable patients, immediate electrical cardioversion or defibrillation is indicated. For stable patients, first-line therapy consists of a 2 g intravenous magnesium bolus, followed by a continuous infusion, aiming for a serum magnesium level greater than 2 mg/dL. Magnesium serves as a critical cofactor for sodium-potassium ATPase activity, which is essential for maintaining normal cardiac conduction [2].

QTc prolongation is frequently observed in patients receiving methadone; however, it is not clear if this is dose dependent [3]. EKG monitoring is advised for patients receiving higher methadone doses, particularly those with additional risk factors for QTc prolongation [1-3]. Although the exact mechanism by which methadone prolongs the QTc is not fully understood, it is believed to involve inhibition of cardiac potassium channels, particularly the delayed rectifier current (IKr), a mechanism also implicated in the development of TdP [1-4]. If TdP persists despite magnesium administration, guidelines from the American College of Cardiology (ACC) and American Heart Association (AHA) recommend initiating isoproterenol, a nonselective β-adrenergic agonist, titrated to maintain a heart rate near 100 beats per minute. Increasing the heart rate shortens the QT interval and reduces the likelihood of premature beats occurring before repolarization is complete. Isoproterenol may terminate TdP or serve as a temporary bridge for overdrive pacing when indicated [4-6].

This article was previously presented as a patient case poster at the CPNP 2022 Annual Meeting (now known as the American Association of Psychiatric Pharmacists (AAPP) Annual Meeting) on April 25th, 2022.

## Case presentation

A 32-year-old female presented to the emergency department with concerns of a possible alcohol withdrawal seizure. During evaluation, she reported taking methadone 190 mg daily for a history of methamphetamine and opioid use disorder. Her past medical history included anxiety, chronic alcohol abuse, a childhood seizure disorder, and tobacco use. Her home medications consisted of methadone, chlordiazepoxide, clonidine, thiamine, and a multivitamin, prescribed during a prior admission for alcohol withdrawal management. Methadone and chlordiazepoxide had been administered approximately eight hours prior to her arrival.

The patient stated that her last alcohol intake occurred at approximately 0900 the day before presentation. Her mother witnessed the seizure and described it as tonic-clonic. The patient’s last known seizure had occurred five years earlier. She also reported symptoms of nausea, vomiting, anxiety, tremors, and headache. An alcohol withdrawal assessment indicated that she was in mild withdrawal.

**Figure 1:**
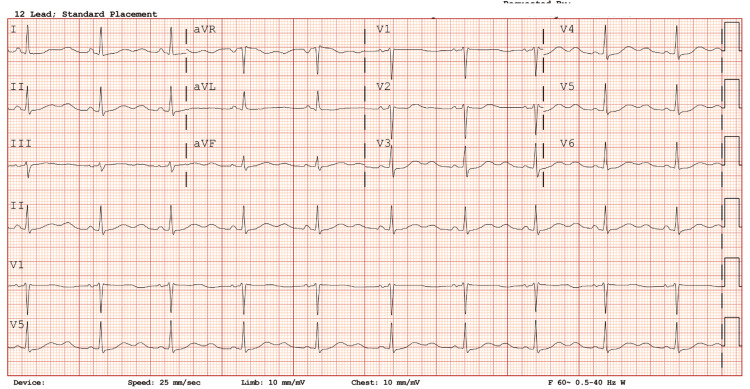
Baseline EKG strip. Baseline rhythm strip obtained on admission. QTc reported as 486 ms.

**Figure 2:**
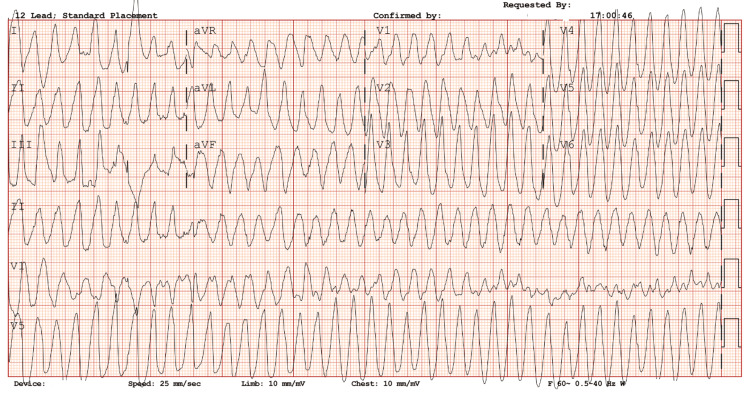
Torsades de pointes. Telemetry strip demonstrating polymorphic ventricular tachycardia consistent with torsades de pointes, characterized by cyclical variations in QRS amplitude (“twisting of the points”). QTc reported as 580 ms.

On presentation, her baseline QTc measured 486 ms (Figure 1), and baseline electrolytes were mildly decreased (Table 1). She was admitted for inpatient management of alcohol withdrawal, and her home medications, including methadone 190 mg once daily and clonidine 0.2 mg twice daily, were continued. She also received one dose of ondansetron 4 mg for nausea/vomiting symptoms. The following day, she developed TdP lasting approximately six minutes, confirmed by EKG monitoring with a QTc of 580 ms (Figure 2). Magnesium 2 grams and lidocaine 100 mg were administered via IV push, successfully terminating the arrhythmia. She was transferred to the intensive care unit, where she continued to experience episodes of TdP.

**Table 1:** Heart rate, electrolyte, and QTc at baseline and during torsades de pointes. Heart rate (bpm); serum magnesium (mg/dL); potassium (mmol/L); QTc (milliseconds).

Date	8/2	8/3
Heart rate (range)	53-70	51-119
QTc	486	580
Potassium	3.4	4.1
Magnesium	1.8	2.3

**Figure 3:**
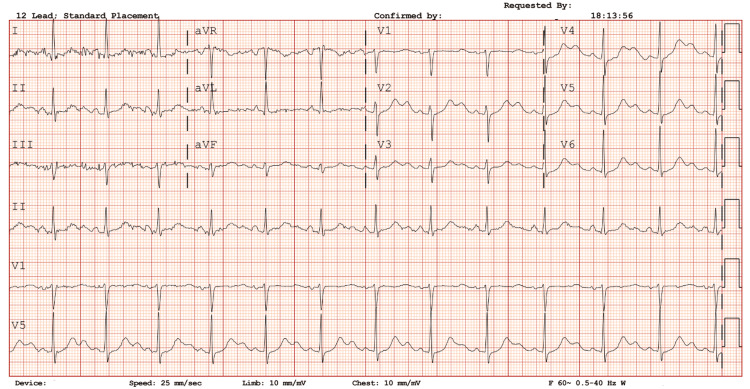
Post-isoproterenol drip. Following discontinuation of offending agents and initiation of isoproterenol (titrated to maintain heart rate of ~90-100 bpm), recurrent torsades de pointes was suppressed.

Cardiology recommended discontinuing methadone and clonidine and initiating isoproterenol along with a magnesium infusion. Isoproterenol was prepared as 2 mg in 500 mL (4 mcg/mL), started at 0.5 mL/hr (0.03 mcg/min) and titrated to 15 mL/hr (1 mcg/min) to maintain a heart rate of approximately 90-100 beats/min; the infusion was briefly increased to 30 mL/hr (2 mcg/min) before being decreased. Following initiation of isoproterenol, no further TdP occurred (Figure 3).

**Figure 4:**
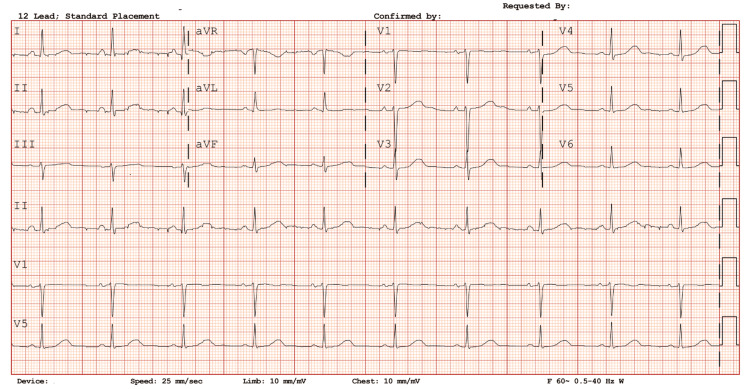
Prior to discharge. Rhythm strip prior to discharge showing QTc improvement (reported 482 ms) with no further torsades de pointes episodes noted four days from the initial event.

After one day, both infusions were weaned off in preparation for a cardiac catheterization. Her QTc subsequently improved to 502 ms and then to 482 ms (Figure 4), and electrolytes were acceptable (Table 2) at discharge. She was referred for genetic testing to evaluate for congenital long QT syndrome, but declined. No further episodes of TdP occurred, and she was discharged after a seven-day hospital stay. Post-discharge EKG follow-up was not available.

**Table 2:** Heart rate, electrolyte, and QTc at resolution of torsades de pointes and day of discharge. Heart rate (bpm); serum magnesium (mg/dL); potassium (mmol/L), and QTc (milliseconds).

Date	8/4	8/7
Heart rate (range)	63-108	54-81
QTc	486	482
Potassium	3.5	4.5
Magnesium	2.9	1.9

## Discussion

This case highlighted multiple risk factors that contributed to prolonged QTc and TdP in a young female maintained on high-dose methadone (Table 3). Her vulnerability was heightened by female sex, a daily methadone dose of 190 mg, concomitant use of a bradycardia-inducing agent, hypokalemia, and acute alcohol withdrawal, factors that collectively increased her risk for QTc prolongation and TdP. Observational data suggest that higher methadone doses are associated with more pronounced QTc prolongation and a greater likelihood of TdP, reinforcing current recommendations for ECG monitoring and consideration of dose reduction or discontinuation when the QTc is markedly prolonged [1-5].

**Table 3:** Risk factors for torsades de pointes (TdP) in this case. The table summarizes risk factors present in this patient during hospital admission. Unknown = not documented.

Risk factor	Present?	Case details
QTc >500 ms	√	580 ms
High-dose QT-prolonging drug	√	Home methadone 190 mg
Additional QT-prolonging drug	√	Ondansetron (single dose)
Female sex	√	32-year-old female
Heart disease	?	Unknown
Congestive heart failure	?	Unknown
Advanced age	—	—
Hypokalemia	√	3.4, mild
Hypomagnesemia	√	1.8, mild
Hypocalcemia	—	Calcium normal
Impaired hepatic drug metabolism	√	Liver impairment
Bradycardia	√	Bradycardia/atrioventricular nodal blockade (e.g., clonidine)

Most documented cases of methadone-associated TdP resolve following methadone discontinuation, electrolyte correction, and intravenous magnesium administration, with occasional use of adjunctive lidocaine [3-5]. In this patient, however, TdP persisted despite these interventions, and telemetry revealed clear pause dependence. Current guidelines for acquired long QT syndrome recommend isoproterenol infusion or temporary transvenous pacing to elevate heart rate and shorten repolarization when TdP is pause-dependent and refractory to magnesium therapy [3-6]. Isoproterenol enhances adrenergic tone and increases sinus rate, thereby suppressing early afterdepolarizations that contribute to TdP in the context of prolonged repolarization.

Acute alcohol withdrawal likely contributed to repolarization instability in this patient. Studies involving individuals with alcohol use disorder have shown that transient QTc prolongation and T-wave abnormalities are common during withdrawal and generally resolve as the withdrawal state improves [7,8]. During alcohol withdrawal, fluctuations in sympathetic tone and electrolyte disturbances can heighten the risk of TdP, even when methadone dosing remains unchanged. In this case, acute withdrawal was layered on top of chronic methadone therapy and clonidine-induced bradycardia, compounding the risk of TdP. Clinicians should recognize this interaction and maintain a low threshold for electrocardiographic monitoring when high-dose methadone use coincides with significant alcohol withdrawal.

**Figure 5:**
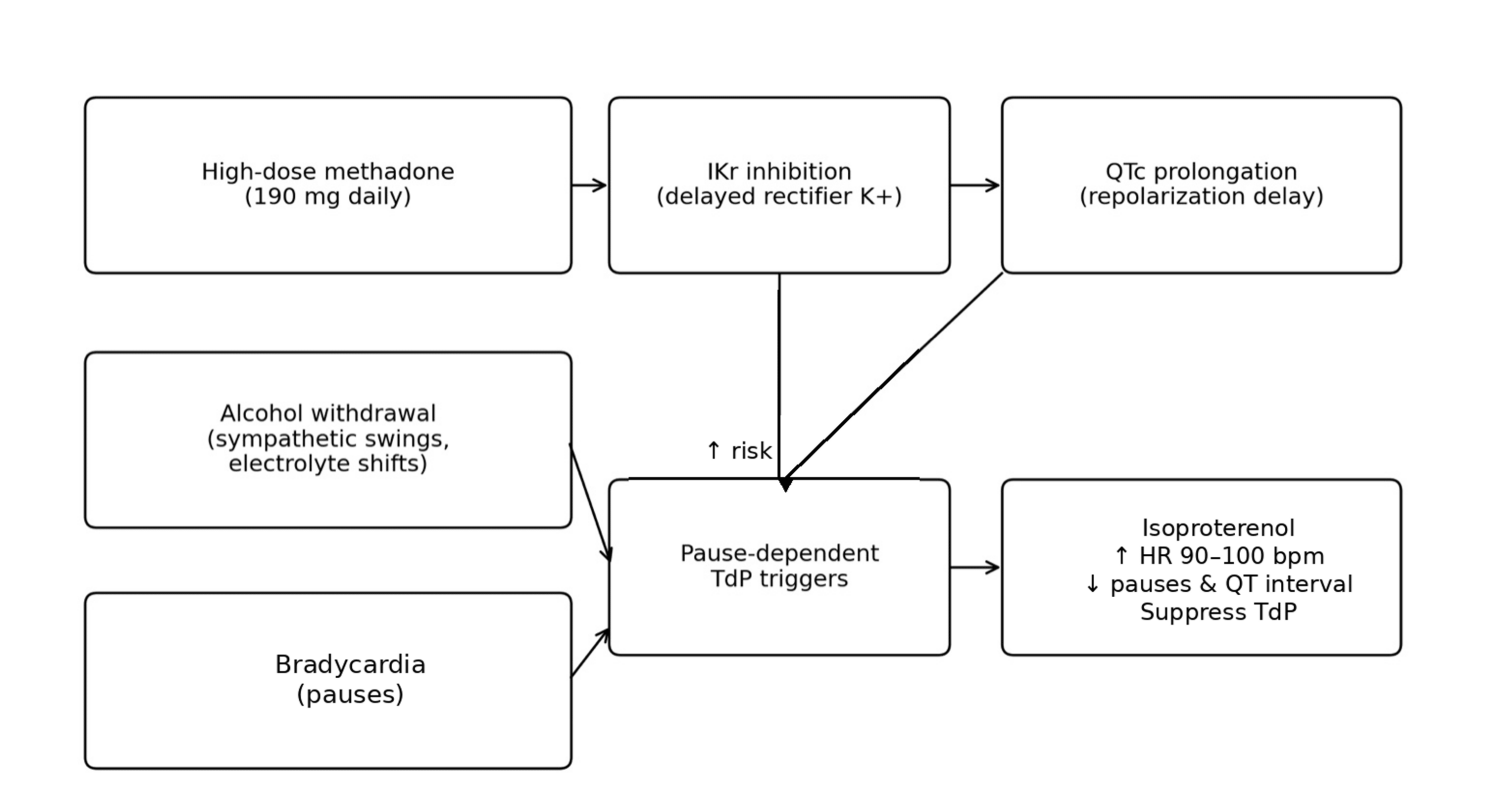
Getting de pointes across: a case report Schematic overview of methadone-associated QTc prolongation, additive risk factors (acute alcohol withdrawal and bradycardia/pause dependence), development of torsades de pointes, and the therapeutic rationale for heart-rate augmentation with isoproterenol.

This case contributes to the limited literature on isoproterenol use in methadone-associated TdP and offers several practical insights (Figure 5). Clinicians should routinely evaluate QTc and associated risk factors in patients receiving high-dose methadone, especially women and those with concurrent bradycardia or undergoing alcohol withdrawal. If TdP persists despite removal of precipitating factors and appropriate magnesium therapy, early implementation of heart rate-accelerating interventions may prevent the need for emergent pacing. Multidisciplinary collaboration among cardiology, critical care, and addiction medicine teams is essential to balance arrhythmia prevention with the continuation of opioid agonist therapy.

Limitations

As a single case report, this observation demonstrates temporal association and physiologic plausibility but cannot establish causality. In addition, outpatient follow-up electrocardiography after discharge was not available at the time of writing.

## Conclusions

QTc prolongation and TdP are known adverse events that can occur in patients receiving high-dose methadone. In this case, alcohol withdrawal, hypokalemia, and a clonidine-associated bradycardia likely amplified her risk. Standard measures, including intravenous magnesium and lidocaine, were insufficient. Isoproterenol titrated to maintain a faster heart rate was associated with prompt suppression of recurrent TdP and progressive QTc improvement. For refractory TdP, isoproterenol is a reasonable option.
